# *Eucommia ulmoides* Polysaccharides Attenuate Rabbit Osteoarthritis by Regulating the Function of Macrophages

**DOI:** 10.3389/fphar.2021.730557

**Published:** 2021-08-06

**Authors:** Yaqiong Sun, Kui Huang, Linhai Mo, Akhlaq Ahmad, Dejia Wang, Zijie Rong, Honghui Peng, Honghua Cai, Guihua Liu

**Affiliations:** ^1^ Departments of Imaging, Southern University of Science and Technology Hospital, Shenzhen, China; ^2^ Departments of Orthopedics, The First Hospital of Yangtze University, Jingzhou, China; ^3^ Department of Orthopaedics, People’s Hospital of Jiangyou, Mianyang, China; ^4^ The Second Affiliated Hospital, Guangdong Provincial Key Laboratory of Allergy & Clinical Immunology, The State Key Laboratory of Respiratory Disease, Guangzhou Medical University, Guangzhou, China; ^5^ Institute of Orthopaedics, Huizhou Municipal Central Hospital, Huizhou, China

**Keywords:** Eucommia ulmoides polysaccharides, immunomodulation, macrophage, osteoarthritis, synovium

## Abstract

**Background and purpose:***Eucommia ulmoides* polysaccharides (EUP) can regulate the immunity of macrophages, but the functional status of macrophages is related to osteoarthritis and synovial inflammation. The purpose of this study is to explore whether EUP has the effect of inhibiting osteoarthritis and its possible mechanism.

**Methods:** MTT test was used to evaluate the appropriate concentration of EUP and real-time quantitative polymerase chain reaction (RT-qPCR) was conducted to detect the effect of EUP on gene expression in RAW 264.7 cells. The osteoarthritis model was constructed by the anterior cruciate ligament transection (ACLT) in the rabbits. These rabbits were divided into three groups, sham operation group, OA group, and EUP group. The changes in articular cartilage were detected by gross observation and histological staining, and Micro-CT tested subchondral bone. Finally, the changes of macrophages in synovial tissue were studied by immunohistochemistry.

**Results:** The results showed that EUP at the concentration of 50ug/mL and 100ug/mL were beneficial to the proliferation of macrophages. The qPCR results indicated that EUP inhibited the expression of inflammation-related genes IL-6, IL-18 and IL-1β, and promoted the expression of osteogenic and cartilage-related genes BMP-6, Arg-1 and transforming growth factor beta (TGF-β). The results of *in vivo* experiments suggested that the degree of destruction of articular cartilage in the EUP group was significantly reduced, and the Osteoarthritis Research Society International (OARSI) score was significantly reduced. Compared with the OA group, the subchondral cancellous bone density of the EUP group increased, the number and thickness of trabecular bone increased, and the separation of trabecular bone decreased. Synovial macrophage immunohistochemistry results manifested that EUP, on the one hand, reduced M1 polarized macrophages, on the other hand, accumulated M2 polarized macrophages.

**Conclusion:** EUP can promote articular cartilage repair and subchondral bone reconstruction. The regulation of the polarization state of macrophages may be one of its mechanisms to delay the progression of osteoarthritis.

## Introduction

Osteoarthritis is a degenerative joint disease, which is mainly manifested as progressive degeneration of articular cartilage, including narrowing of the joint space, changes in subchondral bone structure, osteophyte formation, synovitis etc., (Glyn-Jones et al., 2015). Now more and more evidence shows that synovitis is related to the progression of OA (Sellam and Berenbaum 2010). The normal synovial membrane is composed of the inner membrane layer and the subsynovial layer. When the synovial membrane is inflamed, macrophages accumulate in the inner membrane layer, which is the main pathological feature of synovial inflammation (Udalova et al., 2016). Generally speaking, macrophages can be divided into three different phenotypes according to their sources and functions: unstimulated macrophages (M0), pro-inflammatory macrophages (M1) and anti-inflammatory macrophages (M2) (Xie et al., 2019). Simply put, the M1 type mainly promotes the occurrence of inflammation and can secrete inflammation-related cytokines such as IL-1β, IL-18 and IL-6, which in turn leads to cartilage degradation and the formation of osteophytes, while the M2 type is mainly responsible for anti-inflammatory and tissue repair (Zhang et al., 2020).

**GRAPHICAL ABSTRACT:**
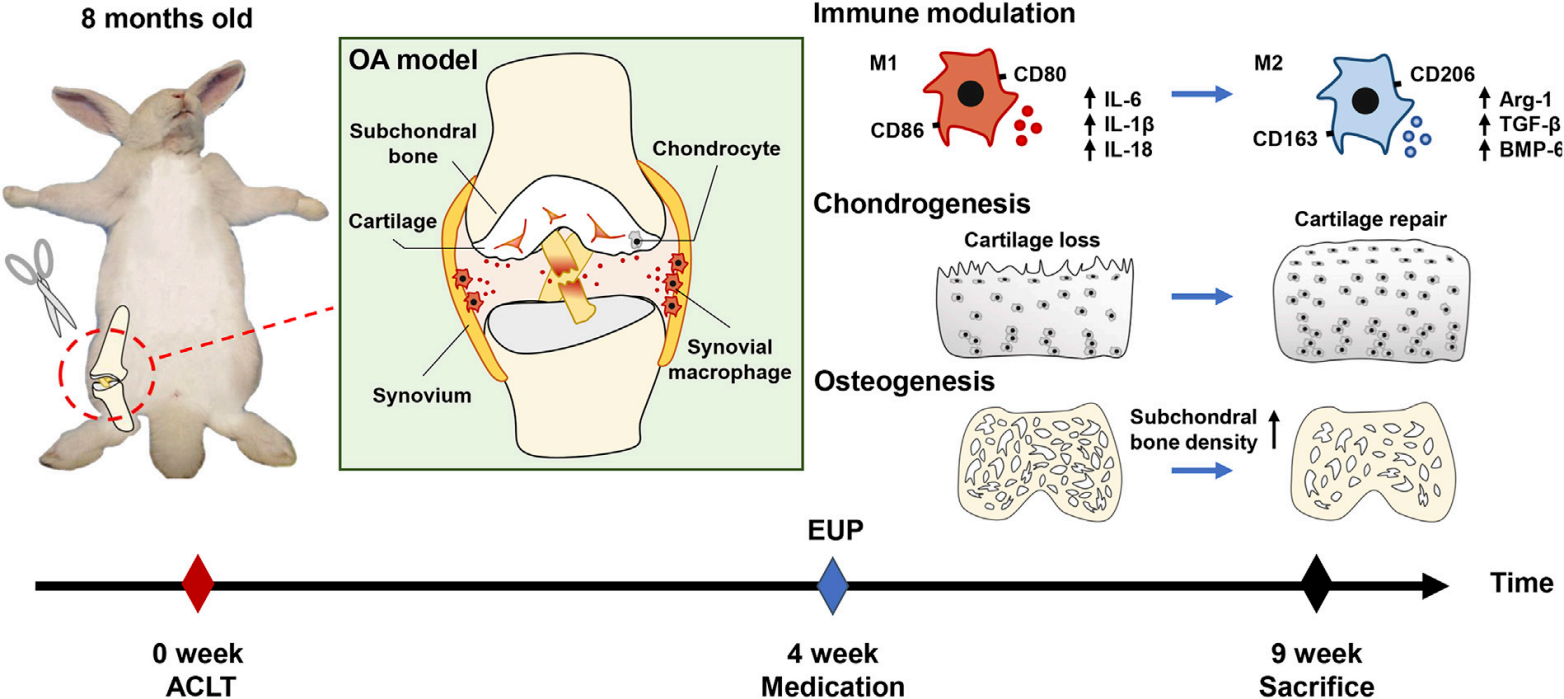


Low-grade inflammation is related to the occurrence and development of osteoarthritis (Robinson et al., 2016). Ayral et al. (2005) conducted a 1 year study on 422 patients and suggested that the degree of inflammation of the synovial membrane is related to the prognosis of OA. (Roemer et al. (2011) showed that joint effusion and synovial inflammation can increase the risk of knee cartilage loss. When joints are injured, local acute inflammation can promote tissue repair, but long-term chronic inflammation is harmful (Oishi and Manabe 2018). Persistent inflammation will promote the production of matrix metalloproteinases (MMPs), leading to degradation of the cartilage matrix (Jiang et al., 2020). Macrophages play an important role in the inflammation process. Studies have shown that low-grade inflammation may not be significantly related to the total number of activated macrophages (Wu et al., 2017), but is related to the polarization state of macrophages (Zhang et al., 2018). This may be the key reason for the continued progression of OA.

Currently, therapies targeting macrophages have shown the ability to reduce inflammation and OA progression. Bondeson et al. (2006) showed that the production of inflammatory cytokines and MMPs can be inhibited by removing macrophages from synovial cell cultures *via* anti-CD14 conjugated magnetic beads. Blom et al. (2004) used liposomal clodronate to deplete synovial macrophages, which significantly reduced osteophyte formation in an animal model of osteoarthritis. *Eucommia ulmoides* is one of the most popular Chinese herbal medicines currently studied. Its main effects are anti-inflammatory, immune regulation and anti-osteoporosis (He et al., 2014). EUP is a component extracted from *Eucommia ulmoides*. It is a powerful immune enhancer (Feng et al., 2016), which can regulate the immune behavior of macrophages and make them develop in the direction of anti-inflammatory. In addition, EUP also has anti-inflammatory and antioxidant properties, as well as inhibits osteoclasts and promotes osteogenic effects (Deng et al., 2019; Gao et al., 2020). In view of the powerful ability of EUP to regulate immunity, it is of great significance to explore its effect on osteoarthritis and its possible mechanism.

This study deeply analyzed the effects and possible mechanisms of EUP in the treatment of osteoarthritis. First, macrophages were processed with EUP *in vitro* and the expression of related genes was detected. Subsequently, the rabbit ACLT model was used to inject EUP into the joint cavity to verify the effect of EUP on osteoarthritis, and the polarization state of macrophages was detected by immunohistochemistry.

## Materials and Methods

### Cell Proliferation Assay

*Eucommia ulmoides* polysaccharides (EUP, purity: 60%, Catalog No.S27810) was obtained from Shanghai Yeyuan biotech Co., Ltd., (Shanghai, China). The RAW 264.7 cell line was purchased from Procell Life Science Technology Co., Ltd. (Wuhan, China). RAW264.7 cells were seeded in 96-well plates with a cell concentration of 2 × 10^3^ cells per well. The culture medium of macrophages is HyClone Dulbecco’s Modified Eagle Medium (DMEM, Gibco) + 5% fetal bovine serum (FBS, Gibco). The RAW264.7 cells were added to DMEM (control group) and DMEM containing different concentrations of EUP (10 μg/ml, 50 μg/ml, 100 μg/ml, 200 μg/ml) and were cultured and recorded under a light microscope. On days 1, 3, and 5, cell proliferation experiments were performed with MTT. The absorbance was measured at 490 nm using a microplate reader (iMark, Bio-Rad).

### Real-Time Quantitative Polymerase Chain Reaction Assay

Macrophages were cultured in DMEM and DMEM + EUP (50 μg/ml, 100 μg/ml) medium for 5 days, and use RNAiso plus (TAKARA) to extract total macrophage RNA according to the instructions provided by the reagent supplier. NanoDrop2000 software was used to determine the total RNA concentration. Then a reverse transcription kit (TAKARA) was used to synthesize cDNA from the obtained total RNA sample. Simply put, 1 μg of total RNA was reverse transcribed at 37°C for 15 min, and then heated at 85°C for 5 s. In the qPCR process, 2 μl of cDNA was used to amplify the target gene. The reactions were run for 40 cycles using a Step one Plus real-time PCR System (Applied Biosystems). Using β-actin as a reference gene, the expression of IL-6, IL18, IL1β, BMP-6, Arg-1, TGF-β was detected. The relative expression of the target genes was calculated using the 2^−ddct^ method with reference to the control group. The primers used for genetic testing are shown in Table 1.

**TABLE 1:** List of primers.

IL-6	F: 5′-TCT​ATA​CCA​CTT​CAC​AAG​TCG​GA-3′
	R: 5′-GAA​TTG​CCA​TTG​CAC​AAC​TCT​TT-3′
IL-18	F: 5′-GAC​TCT​TGC​GTC​AAC​TTC​AAG​G-3′
	R: 5′-CAG​GCT​GTC​TTT​TGT​CAA​CGA-3′
IL-1β	F: 5′-TTC​AGG​CAG​GCA​GTA​TCA​CTC-3′
	R: 5′-GAA​GGT​CCA​CGG​GAA​AGA​CAC-3
BMP-6	F: 5′-GCG​GGA​GAT​GCA​AAA​GGA​GAT-3′
	R: 5′-ATT​GGA​CAG​GGC​GTT​GTA​GAG-3′
Arg-1	F: 5′-CTC​CAA​GCC​AAA​GTC​CTT​AGA​G-3′
	R: 5′-GGA​GCT​GTC​ATT​AGG​GAC​ATC​A-3′
TGF-β	F: 5′-CTT​CAA​TAC​GTC​AGA​CAT​TCG​GG-3′
	R: 5′-GTA​ACG​CCA​GGA​ATT​GTT​GCT​A-3′
β-actin	F: 5′-GGC​TGT​ATT​CCC​CTC​CAT​CG-3′
	R: 5′-CCA​GTT​GGT​AAC​AAT​GCC​ATG​T-3′

### Animal Modeling and Grouping

**FIGURE 1:**
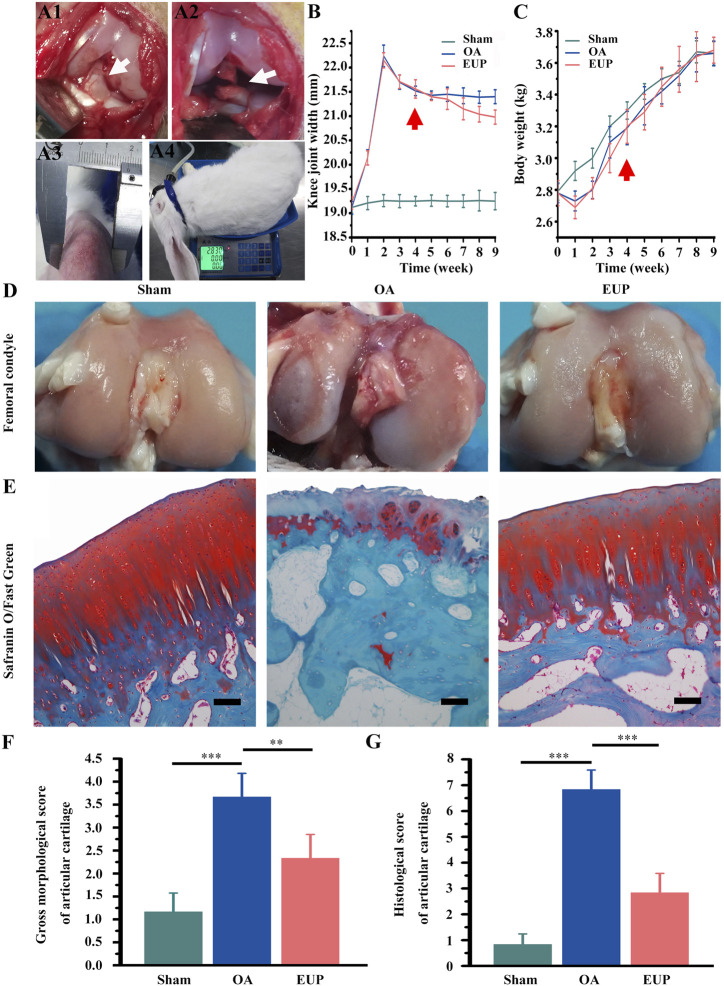
EUP promoted the cartilage repair of rabbit osteoarthritis model constructed by ACLT. **(A1)** The anterior cruciate ligament (ACL) was exposed, the white arrow pointed to the ACL; **(A2)** The arrow showed the ACL was cut off; **(A3)** The width of the rabbit’s knee joint was measured by vernier caliper; **(A4)** Weight measurement. **(B)** The statistical results of the knee joint width, the arrow showed the administration from the 4^th^ week. **(C)** Statistics of rabbit body weight, the arrow showed the administration from the 4 ^th^ week. **(D)** Gross observation of cartilage surface. **(E)** Staining of articular cartilage with Safranin O/Fast Green, bar represents 100 μm. **(F)** Macroscopic morphology evaluation. **(G)** The score of histological staining. **, *** indicate statistically significant differences (*p* < 0.01 and *p* < 0.001, respectively). Bar represents 100 μm.

18 adult male New Zealand white rabbits (2.85 ± 0.2 kg, 8 months old)) were purchased from Experimental Animal Center of Guangdong Medical University (Dongwan, China) and divided into three groups, namely sham operation group, OA group and EUP group. ACLT was performed to establish an OA model. Simply put, after isoflurane inhalation anesthesia, the inside of the knee joint was cut to expose the patella and patellar tendon, and the nodular sac was cut. The anterior cruciate ligament (ACL) was exposed as shown in Figure 1A1 and cut with ophthalmic scissors as shown in Figure 1A2. A drawer test was performed after the operation to confirm the rupture of the ACL. In the sham operation group, only the ACL was exposed and not transected. Three days after the operation, each rabbit was injected intramuscularly with 100,000 units of penicillin every day to prevent infection. Intra-articular injection was started from the 4^th^ week after surgery. The sham operation group and OA group were injected with normal saline, and the EUP group was injected with 100 μg/ml EUP. The injection volume into the joint cavity is 0.1 ml/kg, once a week for five consecutive weeks. At the 9^th^ week, the animals were sacrificed by isoflurane inhalation anesthesia combined with potassium chloride intravenous injection. During the experiment, using vernier caliper and weight scale to measure the width of the knee joints (measure the widest part of the knee) and weight weekly as shown in Figure 1A3 and Figure 1A4.

### Articular Cartilage Repair Test

After rabbit knee articular cartilage was obtained, the degenerative changes of the femoral condyle and tibial plateau cartilage were observed under a dissecting microscope and the severity of OA was scored. The higher the score, the more severe is the cartilage degeneration. The cartilage tissue was fixed with 4% paraformaldehyde for 48 h, and decalcified with 10% EDTA solution for 4 weeks, embedded in paraffin after dehydration. A continuous section with a thickness of 5 μm was prepared, stained with the Safranin O/Fast Green. The histological stained specimens were scored. The above gross and histological scores were based on the methods described in the previous literature (Laverty et al., 2010).

### Micro-CT Measurements

The reconstruction of the femoral condylar bone was observed by micro-CT. Scanning was performed on a micro-CT imaging system of small animals (Skyscan 1276,Bruker). Each sample was scanned layer by layer (scanning thickness 18μm, scanning voltage 80 kV, current 100 μA), and the scanned fault image was obtained. And the quantitative analysis of bone mineral density (BMD) and trabecular thickness, bone trabecular spacing and trabecular number was carried out. BMD was determined by directly calibrating the decay coefficients (250 and 750 mg/cm^3^) of the two hydroxyapatite (HA) models.

### Histological Evaluation of Synovium

The synovium tissue of the inferior patella was fixed with 4% paraformaldehyde for 48 h, and then embedded in paraffin after dehydration. The continuous sections with a thickness of 5 μm were prepared. The SP (Streptavidin perosidase) method was used and SP immunohistochemistry kit was purchased from Beijing boason Biotechnology Co., Ltd., (Beijing, China). In short, after conventional dewaxing, the tissue sections were incubated in 3% hydrogen peroxide in 37°C for 10 min, put into citric acid buffer (pH6.0), boiled (95°C, 15 min), and cooled for 20 min. The normal goat serum working fluid was closed in 37°C for 10 min. The anti F4/80 (1:100, ab16911, Abcam), iNOS (1:100, ab49999, Abcam) and CD206 (1:100, ab8918, Abcam) were all purchased from Abcam company. The secondary antibodies labeled with biotin were incubated overnight at 4°C and incubated at room temperature for 1 h. Then, the streptavidin labeled with horseradish peroxidase was added and incubated at 37°C for 30 min. Finally, DAB was stained for 10 min.

### Statistical Analysis

SPSS 19.0 software package was used for statistical analysis. All results were expressed as mean ± SD. The difference between the two groups was tested by *t*-test. A one-way analysis of variance (ANOVA) was used in the comparison among the groups, *p* < 0.05 was considered to be significant.

## Result

### Macrophage Proliferation and Gene Expression

**FIGURE 2:**
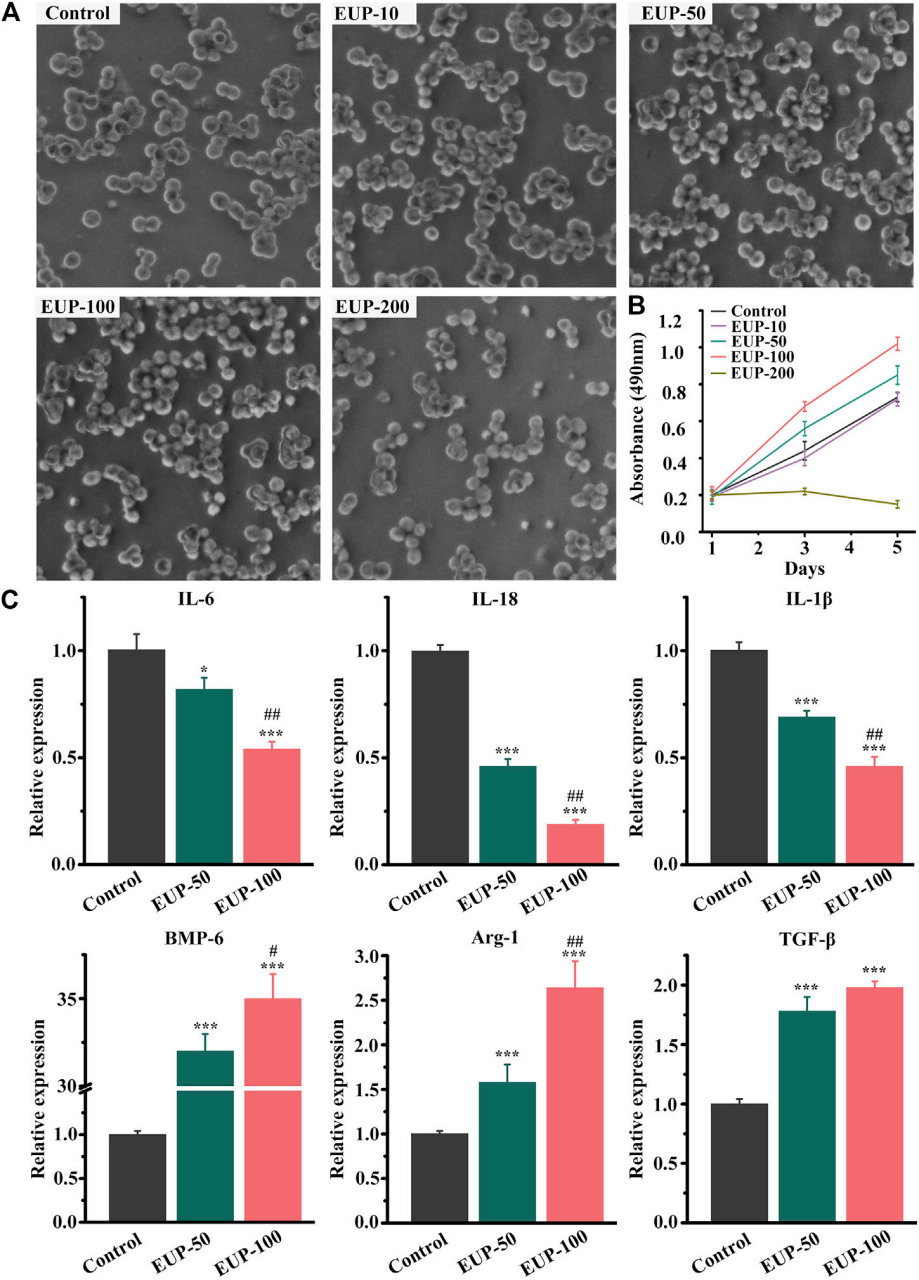
Cell viability and gene expression of macrophages in *Eucommia ulmoides* polysaccharides (EUP) culture. **(A)** The morphology of macrophages in different concentrations of EUP culture. **(B)** The proliferation curve of macrophages was observed under different concentration culture conditions. **(C)** The relative expression of related genes in EUP culture. *p<0.05,***p<0.001 stands for significant difference compared with the control group. ^#^ *p*<0.05,^##^ *p*<0.01 stands for significant difference compared with the EUP-50 group.

Generally, macrophages are round or oval, and they can be multi-process when they are active. Figure 2A showed the status and quantity of macrophages in different concentrations. The macrophages were better than those in control group when the concentration was less than 100 μg/ml. When the concentration was 200 μg/ml, the number of cells decreased and the cells were sparse. MTT results also showed that the concentration of EUP was 200 μg/ml, which inhibited the proliferation of cells. At concentrations of 50 μg/ml and 100 μg/ml, there was no cytotoxicity instead cell proliferation was promoted, as shown in Figure 2B. According to the MTT results, the macrophages were cultured with 50 μg/ml and 100 μg/ml concentration, and the expression of related genes was detected. As shown in Figure 2C below, the relative expression of IL-6, IL-18 and IL-1β decreased significantly. The relative expression of BMP-6, Arg-1 and TGF-β increased.

### Effect of Cartilage Repair in Osteoarthritis After *Eucommia ulmoides* Polysaccharides Treatment

The procedure was shown in Figure 1A1 (exposing the ACL) and 2A2 (cutting the ACL). The changes of joint width and weight were measured after operation, as shown in Figures 1A3, A4. There was no significant change in knee joint width in sham operation group, but in OA group and EUP group, it increased significantly in 4 weeks after ACLT. After injection of EUP joint cavity, the joint width of the EUP group decreased significantly compared with that of OA group as indicated in Figure 1B. In Figure 1C, the weight of OA group and EUP group decreased significantly in 1–2 weeks after operation than that of sham operation group, but there was no significant change in weight between groups over time. As revealed in Figure 1D, the articular cartilage surface of the sham operation group was complete, smooth and glossy. It was indicated that only opening the capsule would not cause the destruction of articular cartilage. The cartilage surface of OA group was brown, damaged and had edema. The cartilage in the EUP group had slight edema and wear, but there was no obvious damage to the integrity of cartilage. The morphological score of the EUP group was significantly lower than that of OA group (*p* < 0.01) (Figure 1F). The results of the Safranin O/Fast Green staining were shown in Figure 1E. The cartilage matrix was dyed red. The cartilage matrix of OA group was destroyed obviously, and the content of cartilage matrix was lower. There was a significant difference between OA group and sham operation group (P< 0.001) (Figure 1G). Compared with OA group, the cartilage matrix in EUP group gradually recovered, and the cartilage thickness was close to normal level. There was significant difference between the EUP group and OA group (P< 0.01).

### Subchondral Bone Reconstruction in Early Osteoarthritis After *Eucommia ulmoides* Polysaccharides Treatment

**FIGURE 3:**
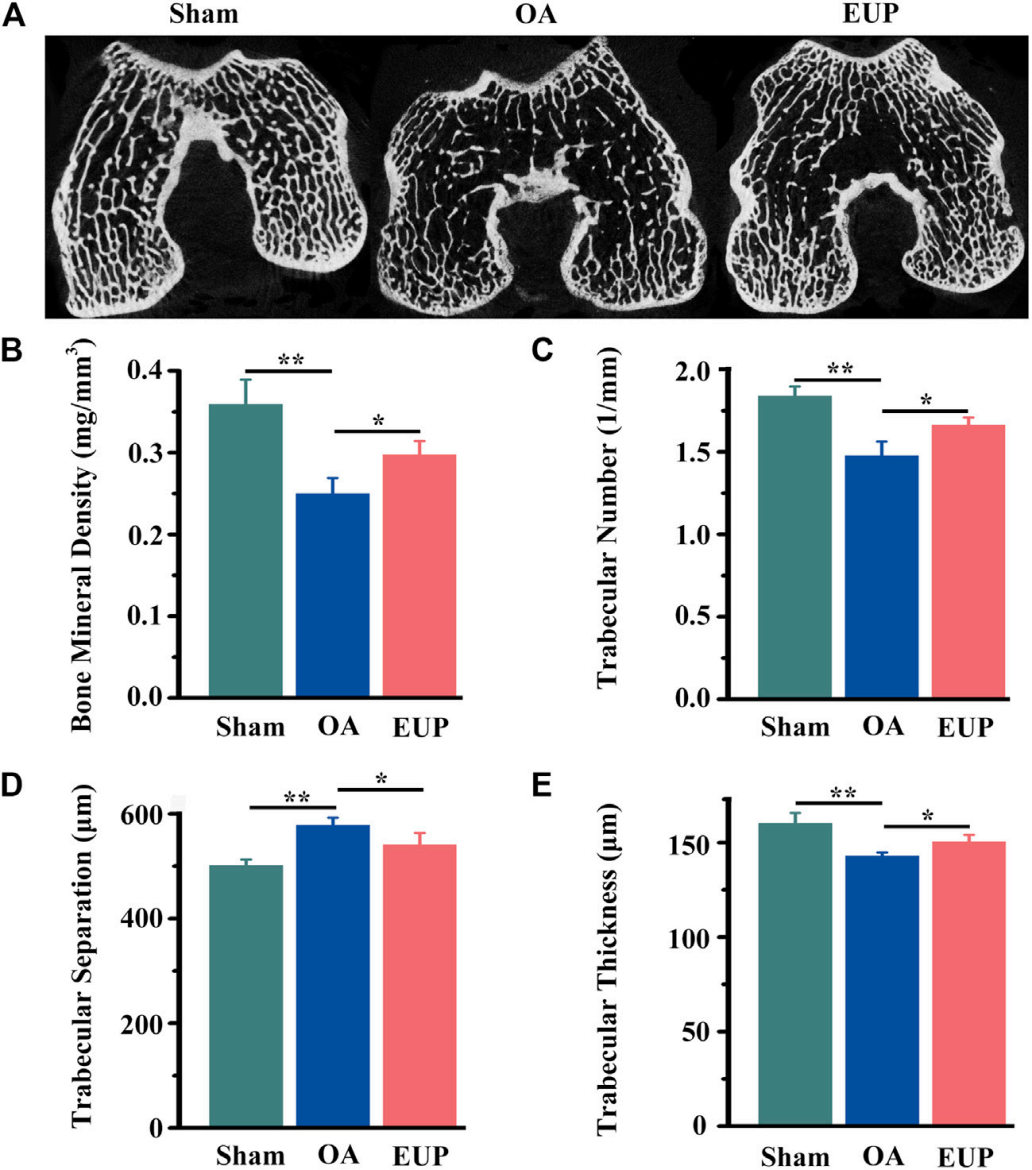
EUP was beneficial to the reconstruction of subchondral bone in early osteoarthritis. **(A)** Micro-CT scan of the subchondral bone of the femoral condyle. **(B)** The BMD of trabecular bone of subchondral bone. **(C)** The number of trabecular bone. **(D)** The separation of trabecular bone. **(E)** The thickness of trabecular bone. *, ** indicate statistically significant differences (*p* < 0.05 and *p* < 0.01, respectively).

In order to further test the effect of EUP on subchondral bone. We used Micro-CT to scan the subchondral bone and perform data analysis. As demonstrated in Figure 3A, the trabeculae of the sham operation group were aligned normally, and no obvious cavities were found. In the OA group, the trabeculae were sparse, arranged disorderly, and cavities were visible. The EUP group was more compact and neatly arranged than the OA group. Quantitative statistics suggested that the BMD of trabecular bone in the OA group was significantly lower than that in the sham operation group (*p* < 0.01), but the BMD of the EUP group was higher than that in the OA group (P< 0.05) (Figure 3B). The number of trabecular bone (Figure 3C) and the thickness of trabecular bone (Figure 3E) in the EUP group were better than those in the OA group (P< 0.05). The separation of trabecular bone in the OA group was higher than that in the sham operation group, and the separation of trabecular bone in the EUP group was lower than that in the OA group (P< 0.05) (Figure 3D).

### Effect of Synovial Macrophage After *Eucommia ulmoides* Polysaccharides Treatment

**FIGURE 4:**
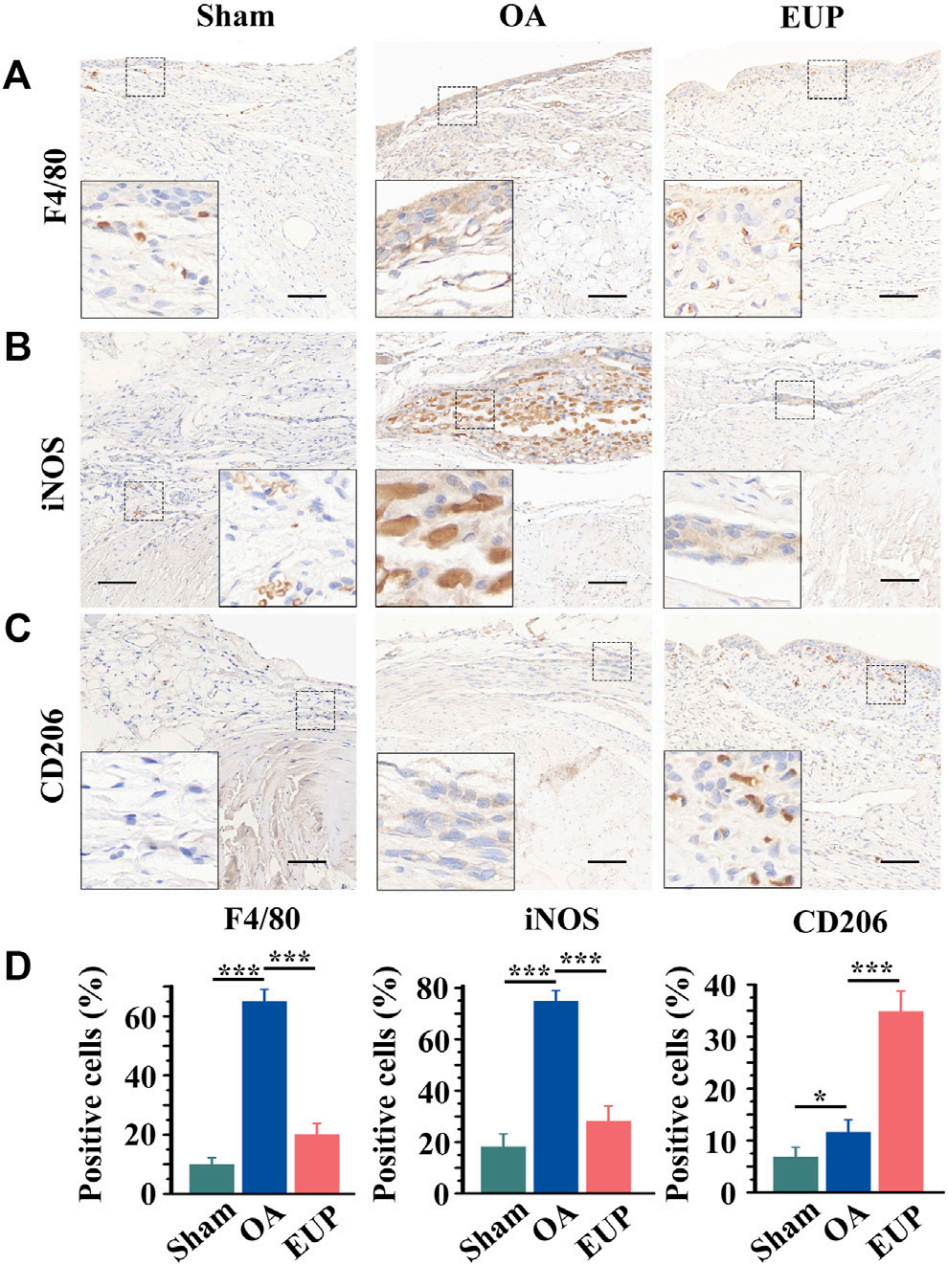
Changes in the immunophenotype of macrophages in synovial tissue after EUP treatment. **(A)** immunohistochemistry of F4/80. **(B)** immunohistochemistry of iNOS. **(C)** immunohistochemistry of CD206. bar represents 100 μm. **(D)** Quantitative detection of the proportion of F4/80, iNOS and CD206-positive macrophages in total macrophages. *, *** indicate statistically significant differences (*p* < 0.05 and *p* < 0.001, respectively).

The possible mechanisms were explored by detecting the immunophenotype of macrophages in synovial tissues. F4/80 is a marker of macrophages, iNOS is a marker of M1-like macrophages, and CD206 is a marker of M2-like macrophages. As shown in Figures 4A,B, compared with the sham group, F4/80 positive cells and iNOS positive cells were significantly increased in the OA group. Compared with the OA group, the number of F4/80 and iNOS positive cells in the EUP group decreased. However, CD206 positive cells were significantly increased in the EUP group (Figure 4C). As demonstrated in Figure 4D, the expression of F4/80 and iNOS positive cells was significantly higher in the OA group than in the sham group and the EUP group. The expression of CD206 positive cells in the EUP group was significantly higher than that in the sham group and OA group, and the expression of CD206 positive cells in the OA group was higher than that in the sham group.

## Discussion

The monosaccharide components of EUP include glucose, fructose, mannose, fucose, galactose, and arabinose among others (Feng et al., 2016). Sugars themselves provide nutrients for cell growth. Studies have shown that cell proliferation is highly dependent on glucose metabolism, which not only provides an energy source but also provides metabolites for the biosynthesis of membrane lipids and nucleic acids (Shao et al., 2018). Our experiments also indicated that at 50μg/ml and 100μg/ml concentrations, EUP was not cytotoxic but rather favored cell proliferation.

Through PCR assay, we found that EUP significantly inhibited the expression of IL-6, IL-18 and IL1β. Studies have shown that the cytokine IL-6, IL-1β and IL-18 can promote inflammation and apoptosis of chondrocytes, they inhibit the expression of aggrecan and stimulate the expression of MMPs, thus contribute to the pathogenesis of OA (Bao et al., 2020; Kapoor et al., 2011). Therefore, inhibiting the expression of inflammation related genes IL-6, IL-1 β, and IL-18 is beneficial for the remission of osteoarthritis. Increased Arginase-1 (Arg-1), TGF-β and BMP-6 expression was found. Arg-1 expression is one of the characteristics of M2 macrophages, which are anti-inflammatory and promote tissue repair (Fernandes et al., 2020). The expression of TGF-β in macrophages is pro regenerative (Dai et al., 2018). BMP-6 is closely related to osteogenesis (Huang et al., 2019). So the results suggested that EUP may favor macrophage polarization toward M2 type direction and be beneficial to the regeneration of cartilage and osteogenesis.

In this study, we found that the rabbit knee joint widens after cutting the ACL. This may be related to inflammation of the joint cavity and increased joint cavity effusion (Bian et al., 2018). Compared with the OA group, the joint width of the EUP group decreased significantly after the third week after treatment, indicating that the EUP controlled the inflammation of the joint cavity and reduced joint effusion. the injection of EUP through the joint cavity of OA model showed that cartilage repair was significantly improved in the EUP group, and gross and histology scores showed that the EUP group was significantly reduced. Our experiments at the cellular level have suggested that EUP inhibits inflammation in macrophages and promotes macrophage polarization toward the M2 type direction. Therefore, we think that EUP may promote cartilage repair in osteoarthritis by regulating macrophages.

Our results indicated that the BMD of trabecular bone significantly increased, the separation of trabecular bone became smaller and the number of trabecular bone increased in EUP group. According to qPCR results as previously described that EUP promoted Arg-1 and BMP-6 expression in macrophages. Hoemann et al. (2010) showed that activated macrophages expressed Arg-1, released angiogenic factors, and thus contributed to the repair of subchondral bone. BMP-6 had a significant pro-osteogenic effect (Grab et al., 2019). The promotion of subchondral bone remodeling will contribute to cartilage regeneration and the recovery of joint function (Hayami et al., 2006). Furthermore, in conclusion, EUP also benefit the reconstruction of subchondral bone.

The results of immunohistochemistry demonstrated that EUP inhibited the expression of macrophages and M1-like macrophages, and increased the expression of M2-like macrophages. As mentioned earlier, in our experiment, EUP promotes the expression of Arg-1 in macrophages. The transformation of macrophages from pro-inflammatory M1 to M2 may be related to EUP promoting the expression of Arg-1. Luo et al. (2020) demonstrated that macrophage polarization switch from proinflammatory M1 type to M2 type is an effective therapeutic strategy for the treatment of temporomandibular arthritis. Taken together, the above results suggest that EUP may alleviate osteoarthritis and promote cartilage repair by regulating macrophage function and promoting their polarization towards M2 type.

## Conclusion

In this study, we found that EUP exhibited no obvious toxicity to cells at the concentrations of 50μg/ml and 100μg/ml, but favored cell proliferation. qPCR assay indicated that EUP significantly inhibited the expression of macrophage inflammation related genes IL-6, IL-18 and IL-1β and promoted the expression of osteogenesis and chondrogenesis related genes BMP-6, Arg-1 and TGF-β. *In vivo* detection found that the cartilage regeneration was significantly improved, and micro-CT scanning found that EUP was beneficial to subchondral bone reconstruction. Examination of synovial macrophages revealed that the number of macrophages and M1 type macrophages decreased and the number of M2 type macrophages increased in the EUP group compared with the OA group. Through our experimental results, we believe that EUP can delay the progression of osteoarthritis and the effect of regulating the immunity of macrophages partly explains the underlying mechanism.

## Data Availability

The raw data supporting the conclusion of this article will be made available by the author, without undue reservation.
